# Interplay between miR27a and PPARγ–DUSP1 regulates BPXV mRNA translation via modulating p38α MAPK–MNK1–eIF4E signaling pathway

**DOI:** 10.1016/j.virusres.2026.199811

**Published:** 2026-09-29

**Authors:** Garvit Kumar, Assim Verma, Shweta Dhanda, Himanshu Kamboj, Riyesh Thachamvally, Shoor Vir Singh, Ram Kumar, Naveen Kumar

**Affiliations:** a National Centre for Veterinary Type Cultures, ICAR-National Research Centre on Equines, Hisar, India; b Department of Biotechnology, GLA University, Mathura, India; c ICMR-National Institute of Virology, Pune, India

**Keywords:** PPARγ, miR-27a, p38α MAPK, Poxvirus, BPXV

## Abstract

In this study, we demonstrate that activation of PPARγ (Peroxisome Proliferator-Activated Receptor Gamma), a nuclear receptor and transcription factor, by its agonist Rosiglitazone significantly suppresses replication of Buffalopox virus (BPXV). Time-of-addition and step-specific assays revealed that rosiglitazone primarily inhibits viral genome replication and protein synthesis, while showing no measurable effect on viral attachment, entry, or release in infected cells. Mechanistic investigations identified miR-27a, an endogenous negative regulator of PPARγ, as a proviral factor displaying an inverse expression pattern with PPARγ during infection. At 12 h post-infection, corresponding to the peak of viral mRNA translation, miR-27a expression was maximal whereas PPARγ levels were markedly reduced, suggesting a critical regulatory axis controlling viral translation. Activation of PPARγ suppressed the p38α MAPK–MNK1–eIF4E signaling pathway required for cap-dependent viral mRNA translation. Notably, the antiviral effect of rosiglitazone was abolished in p38α-knockout cells, confirming the involvement of p38α signaling. Although PPARγ did not directly interact with p38α, it induced the expression of DUSP1, a known negative regulator of p38α activity. Pharmacological inhibition of DUSP1 restored viral replication in rosiglitazone-treated cells, supporting a PPARγ–DUSP1-mediated mechanism. Furthermore, rosiglitazone significantly reduced BPXV-induced mortality and pock lesion formation on the chorioallantoic membrane of specific pathogen-free embryonated chicken eggs. Importantly, prolonged viral passage in the presence of the inhibitor did not lead to the emergence of resistant variants, underscoring the potential of rosiglitazone as a host-directed antiviral strategy with a low likelihood of inducing antiviral resistance.

## Highlights

• This study provides the first evidence for the involvement of PPAR-γ in Buffalopox virus (BPXV) replication.
• Activation of PPAR-γ by rosiglitazone significantly suppresses BPXV replication, indicating its antiviral potential.
• In contrast, miR-27a promotes BPXV replication, demonstrating a pro-viral role during infection.
• This study provides the first mechanistic insight into the role of the PPARγ–DUSP1–p38α–MNK1–eIF4E signaling axis in regulating BPXV protein translation.
• Rosiglitazone treatment does not generate drug-resistant BPXV variants, even after serial viral passages.

## Graphical abstract

**Figure:**
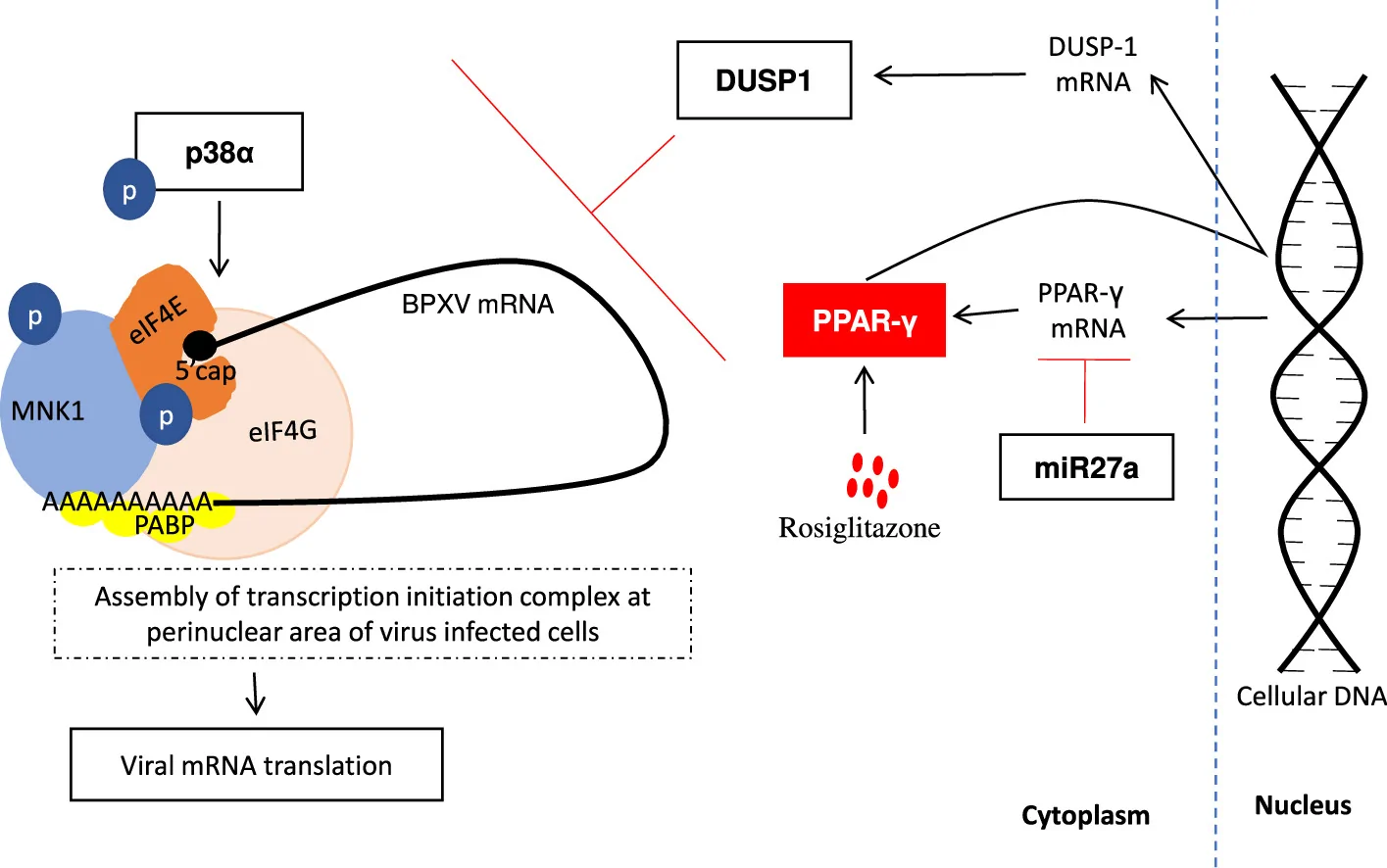

## 1 Introduction

Buffalopox virus (BPXV), a zoonotic *Orthopoxvirus*, poses significant threats to both livestock and public health. This challenge is compounded by the lack of effective vaccines and antiviral therapies (Kumar et al., 2018a). As obligate intracellular parasites, viruses rely completely on host cellular machinery for their replication, interacting with host factors that can either promote (proviral) or inhibit (antiviral) various stages of their life cycle (Khandelwal et al., 2020). Targeting these host factors rather than viral components, presents a promising strategy for the development of broad-spectrum antiviral agents. Such approaches are less prone to antiviral drug resistance, since viruses are typically unable to compensate for the loss of essential host functions through mutation (Kumar et al., 2020).

Peroxisome proliferator activated receptor gamma (PPARγ), encoded by the PPARG gene, is a type II nuclear receptor expressed in a wide variety of cells including epithelial cells, fibroblasts, adipose tissue, lymphocytes and macrophages (Yu et al., 2023). It exists in two principal isoforms: PPARγ1, which is ubiquitously expressed across multiple tissues, and PPARγ2, which is predominantly expressed in adipose tissue. Together, these isoforms play key roles in regulating adipogenesis, glucose homeostasis, lipid metabolism, and inflammatory responses (Hu et al., 2022; Lefterova et al., 2014; Liu et al., 2020; Pravin et al., 2025). In metabolic and inflammatory pathways, PPARγ acts as a central node, integrating upstream signals from factors such as miR-27a and p38 MAPK (Chen et al., 2019b; Schild et al., 2006). Under inflammatory conditions, p38 MAPK activation leads to increased expression of miR-27a, which directly binds to the 3′ untranslated region (UTR) of PPARγ mRNA, suppressing its translation (Cao and Chen, 2017; Kim et al., 2010a; Shi et al., 2021). This results in diminished PPARγ activity, with downstream consequences for adipogenesis, glucose homeostasis and inflammatory responses (Sun et al., 2021). Thus, elevated miR-27a and activated p38 MAPK act in concert to reduce PPARγ function, disrupting metabolic balance. Conversely, active PPARγ can in turn inhibit p38 MAPK signaling, demonstrating bidirectional regulation within this axis (Hua et al., 2022).

Previous studies have identified miR-27a as virus restriction in murine cytomegalovirus (MCMV) infection (Buck et al., 2010), and PPARγ as proviral factor for human cytomegalovirus (HCMV) (Rauwel et al., 2010). In contrast, PPARγ was also shown to restrict the growth of HIV-1 and hepatitis B virus (HBV) (Hayes et al., 2002; Potula et al., 2008; Wakui et al., 2010).

The p38 MAPK signaling cascade is also increasingly recognized as a central regulator of replication for many viruses (Kumar et al., 2020). Similar to PPARγ, the role of p38 MAPK is context-dependent; in certain viral infections, it has been shown to promote viral replication, whereas in others, it exerts inhibitory effects (Chander et al., 2022; Kumar et al., 2018b; Mittal et al., 2024; Su et al., 2025). In the case of poxviruses, especially BPXV, p38 MAPK serves as a proviral factor, primarily by supporting the translation of viral mRNAs (Chander et al., 2022). However, besides p38 MAPK, the exact roles of PPARγ and miR-27a in poxvirus, particularly in BPXV, remain largely unexplored. Therefore, in this study, we elucidate the role of cellular interplay between PPARγ, miR-27a, and the p38 MAPK axis in BPXV replication.

## 2 Materials and methods

### 2.1 Chemicals

Rosiglitazone, GW9662, DUSP1 inhibitor, MTT [3-(4,5-dimethyl-2-thiazolyl)-2,5-diphenyl-2H-tetrazolium bromide] and dimethyl sulfoxide (DMSO) were procured from Sigma (Steinheim, Germany).

### 2.2 Antibody

Phospho-P38α Α Rabbit monoclonal antibody, Phospho-MNK1 Rabbit monoclonal antibody, Phospho-eIF4E Rabbit monoclonal antibody, anti-eIF4E mouse monoclonal antibody, P38 MAPK Rabbit monoclonal antibody, MNK1 Rabbit monoclonal antibody, Rhodamine-conjugated anti-rabbit antibody (Invitrogen), Fluorescein isothiocyanate (FITC) conjugated anti- mouse antibody (Sigma), Mouse anti-GAPDH (Glyceraldehyde 3-phosphate dehydrogenase, house-keeping control protein) primary antibody, Anti-Mouse IgG (whole molecule)-Alkaline Phosphatase antibody (produced in goats) and Anti-Rabbit IgG (whole molecule)–Peroxidase antibody (produced in goat)were received from Sigma-Aldrich (St. Louis, USA). Anti-BPXV hyperimmune serum, generated in rabbits, was obtained from NCVTC, Hisar. The production and characterization of this serum have been previously documented.

### 2.3 Cell culture

African green monkey kidney (Vero) cells were obtained from the National Centre for Cell Science (NCCS), Pune, India. The cells were grown in Dulbecco’s Modified Eagle’s Medium (DMEM) supplemented with 5–10% fetal calf serum (FBS) (Sigma, St. Louis, USA) and antibiotics (Chander et al., 2022).

### 2.4 p38α MAPK knockout cell

The p38α MAPK knockout cell, generated by CRISPR/Cas9-mediated genome editing by our group, has described previously (Chander et al., 2022).

### 2.5 Virus

The BPXV strain adapted to Vero cells (Accession Number: VTCC-AVA90) was provided by NCVTC Hisar. The virus was cultured in Vero cells and its concentration was measured by performing a plaque assay as described previously. The viral load was expressed in plaque-forming units per milliliter (PFUs/mL).

### 2.6 Determination of cytotoxicity (MTT assay) and virucidal activity

The cytotoxic and virucidal activity of Rosiglitazone, GW9662 and DUSP1 inhibitor were determined as previously described (Kumar et al., 2022b).

### 2.7 Time-of-addition assay

Vero cell monolayers, in triplicates, were infected with BPXV at an MOI of 5. Rosiglitazone or DMSO (vehicle control) were added at 0.5 h before infection or at 1, 3,6, 12, 18, and 24 h post-infection (hpi). Supernatants were collected at 48 hpi to measure viral titers by plaque assay (Kumar et al., 2022c).

### 2.8 Attachment assay

Cells were pretreated with Rosiglitazone or vehicle control for 1 h, then exposed to BPXV (MOI 5) for 1 h at 4 °C to allow virus binding without entry. After washing to remove unbound virus, cells were lysed by freeze-thaw cycles and the amount of cell-associated virus was quantified by plaque assay.

### 2.9 Entry assay

Vero cells were first incubated with BPXV (MOI 5) at 4 °C for 1 h to permit viral attachment. Following PBS washes, cells were incubated with serum-free medium containing rosiglitazone or DMSO at 37 °C for 1 h to enable viral entry. After removing extracellular virus by washing, cells were maintained in inhibitor-free medium. Viral titers in supernatants collected at 48 hpi were determined by plaque assay.

### 2.10 Virus release assay

Vero cells were infected with BPXV at an MOI of 5 for 1 h, followed by washing thoroughly with PBS to remove any unbound virus. Thereafter, fresh medium was added and the cells were incubated at 37 °C. At 36 hpi, when viral release typically expected to begin, Rosiglitazone or DMSO (vehicle control) were added. Supernatants were collected 1 h and 2 h after drug treatment and the virus titres were measure by the plaque assay.

### 2.11 Quantitation of the mRNA levels (qRT-PCR)

Confluent Vero cell monolayers, in triplicates, were infected with BPXV at an MOI of 5 for 1 h, followed by washing with PBS and addition of fresh DMEM. Rosiglitazone (0.45 µM) or 0.05% DMSO (vehicle control) were added at 6 hpi. Cells were harvested at 12 hpi for nucleic acid extraction (Khandelwal et al., 2017). The RNA was extracted using TRIzol reagent and the complementary DNA (cDNA) was synthesized from RNA using oligo(dT) primers. qRT-PCR was performed to quantify viral (*M gene*) and cellular *(PPARγ gene)* mRNA levels by gene-specific primers (**Table S1**). Relative quantification of viral and cellular mRNA was calculated using the comparative ΔΔCt method, normalizing to GAPDH expression.

### 2.12 Quantitation of BPXV DNA levels (qRT-PCR)

Confluent Vero cell monolayers, in triplicates, were infected with BPXV at an MOI of 5 for 1 h, followed by washing with PBS and addition of fresh DMEM. Rosiglitazone (0.45 µM) or 0.05% DMSO (vehicle control) was added at 6 hpi. Cells were harvested at 36 hpi for nucleic acid extraction (Khandelwal et al., 2017). Total DNA was isolated using standard extraction methods. Viral DNA levels were quantified by qRT-PCR targeting the BPXV membrane protein (M) gene, with GAPDH as the internal reference for normalization. Relative quantification of viral DNA levels was calculated using the comparative ΔΔCt method.

### 2.13 miR27a levels

Total RNA was extracted using TRIzol reagent and concentrations were standardized to 1 µg per sample. Poly(A) tails were added using *Escherichia coli* Poly(A) Polymerase according to the manufacturer’s protocol. Reverse transcription was performed with an oligo adaptor primer to generate cDNA. qRT-PCR was performed using a miR-27a-specific forward primer and a universal adaptor reverse primer (**Table S1**). Expression levels were normalized by U6, a housekeeping control microRNA and relative fold change was calculated by the ∆∆ Ct method.

### 2.14 Viral and host protein expression

Vero cells were infected with BPXV at an MOI of 5 for 1 h. Rosiglitazone or DMSO (vehicle control) was added at 3 hpi, and cells were further incubated at 37 °C. At 36 hpi, cells were harvested by scraping and lysed for Western blot analysis. Viral protein levels were detected using anti-BPXV serum, while glyceraldehyde-3-phosphate dehydrogenase (GAPDH) served as an internal control (Khandelwal et al., 2017). The expression of host proteins, including phosphorylated and total p38α, MNK1, and eIF4E, was determined using their respective primary antibodies.

### 2.15 The proviral activity of miR-27a

The hsa-miR-27a-3p mimic (sequence: 5′-UUCACAGUGGCUAAGUUCCGC-3′), hsa-miR-27a-3p inhibitor (sequence: 5′-GCGGAACUUAGCCACUGUG-3′) and non-targeting negative control mimic were procured from MedChemExpress (Suite F, USA). Transfections were performed at a final concentration of 50 nM, a dose confirmed to be non-toxic by MTT assay, following standard miRNA transfection protocol (Kumar et al., 2024).

### 2.16 Immunoflurescence assay

Vero cells were grown in chamber slides and infected with BPXV at an MOI of 5 for 1 h. Inhibitor was applied at 4 hpi and intracellular localization of BPXV (viral proteins) and host protein (eIF4E) in the virus-infected cells was examined at 36 hpi by immunofluorescence assay. Briefly, the cells were fixed with 4% paraformaldehyde for 15 min and blocked by 2% BSA for 30 min at room temperature. After being washed with PBS, cells were stained with rabbit anti-BPXV hyper-immune serum and anti-eIF4E mouse monoclonal antibody (Sigma) for 1 h in the presence of 0.2% saponin. Cells were washed three times with PBS and incubated with a secondary Rhodamine-conjugated anti-rabbit antibody (Invitrogen) fluorescein isothiocyanate (FITC) conjugated anti- mouse antibody (Sigma) in the presence of 0.2% saponin for 30 min. After being washed again with PBS, the cells were mounted with a medium containing 4,6-diamidino-2-phenylindole (DAPI, Sigma) and examined by fluorescence microscopy.

### 2.17 In ovo antiviral efficacy

***(a) Determination of* LD_50_*of rosiglitazone:*** Ten-day-old specific pathogen-free (SPF) embryonated chicken eggs (Indovax Pvt. Ltd., Hisar, India) were used to determine the median lethal dose (LD_50_) of rosiglitazone. Four fold serial dilutions of rosiglitazone (concentration ranging from 20 to 0.3 µg/egg) or equivalent volumes of DMSO, in 10 days old embryonated SPF eggs, in a total of 100 μL volumes via the CAM route. The survival of the chicken embryos was monitored daily by candling for five days following infection. LD_50_ value was calculated using the Reed-Muench method (Chander et al., 2022).

***(b) Assessment of antiviral efficacy* (EC_50_):** To test the antiviral activity, SPF eggs were infected with BPXV at 100 EID_50_ via the CAM route. Immediately after infection, eggs were treated with rosiglitazone at 0.5, 0.25, 0.12, or 0.07 μg/egg (in 100 μl volume per egg). Vehicle-treated eggs served as controls. At 5–7 days post-infection, eggs were examined for the development of the pock lesions on the CAM and viability of the embryo (Chander et al., 2022). The EC_50_was determined by the Reed-Muench method.

### 2.18 Selection of potential rosiglitazone-resistant virus variant

Vero cells were infected with BPXV at an MOI of 0.1 in medium supplemented with 0.15 µM rosiglitazone or 0.05% DMSO (vehicle control). After 48–72 hpi, supernatants were collected (passage 1, P1) and viral titers were determined by plaque assay. This procedure was repeated for 50 consecutive passages, each time infecting fresh Vero monolayers with supernatant from the previous passage under identical treatment conditions. Following the 50th passage, the original virus stock (P0), rosiglitazone-passaged virus (P50-rosiglitazone), and control-passaged virus (P50-control) were used to infect Vero cells at MOI 0.1 in the presence of 0.45 µM rosiglitazone or DMSO. Viral titers were measured at 48 hpi to evaluate potential changes in viral susceptibility resulting from prolonged exposure to rosiglitazone (Kumar et al., 2024).

## 3 Results

### 3.1 PPARγ antagonize BPXV replication

To evaluate the role of PPARγ signaling in BPXV replication, we employed rosiglitazone **(Fig. S1A)**, a selective PPARγ agonist. At non-cytotoxic concentrations (**Fig. S1B**), rosiglitazone inhibited BPXV replication in a dose-dependent manner (Fig. 1**A**). Importantly, rosiglitazone did not exhibit virucidal activity against cell-free viral particles (Fig. 1**B**), suggesting that its antiviral effect is likely mediated by interference with the viral replication cycle within host cells rather than direct inactivation of extracellular virus. To further confirm the antiviral role of PPARγ, we utilized GW9662, a selective PPARγ inhibitor **(Fig. S1C)**. At a non-cytotoxic concentration (**Fig. S1D**), GW9662 significantly enhanced BPXV replication (Fig. 1**C**), further supporting the notion that activation of PPARγ antagonize BPXV replication. Besides this, rosiglitazone also inhibited replication of Lumpy skin disease virus (LSDV) (Fig. 1D) and vaccinia virus as well (Fig. 1E), suggestive its broad spectrum antiviral effect against poxviruses.

**Fig. 1:**
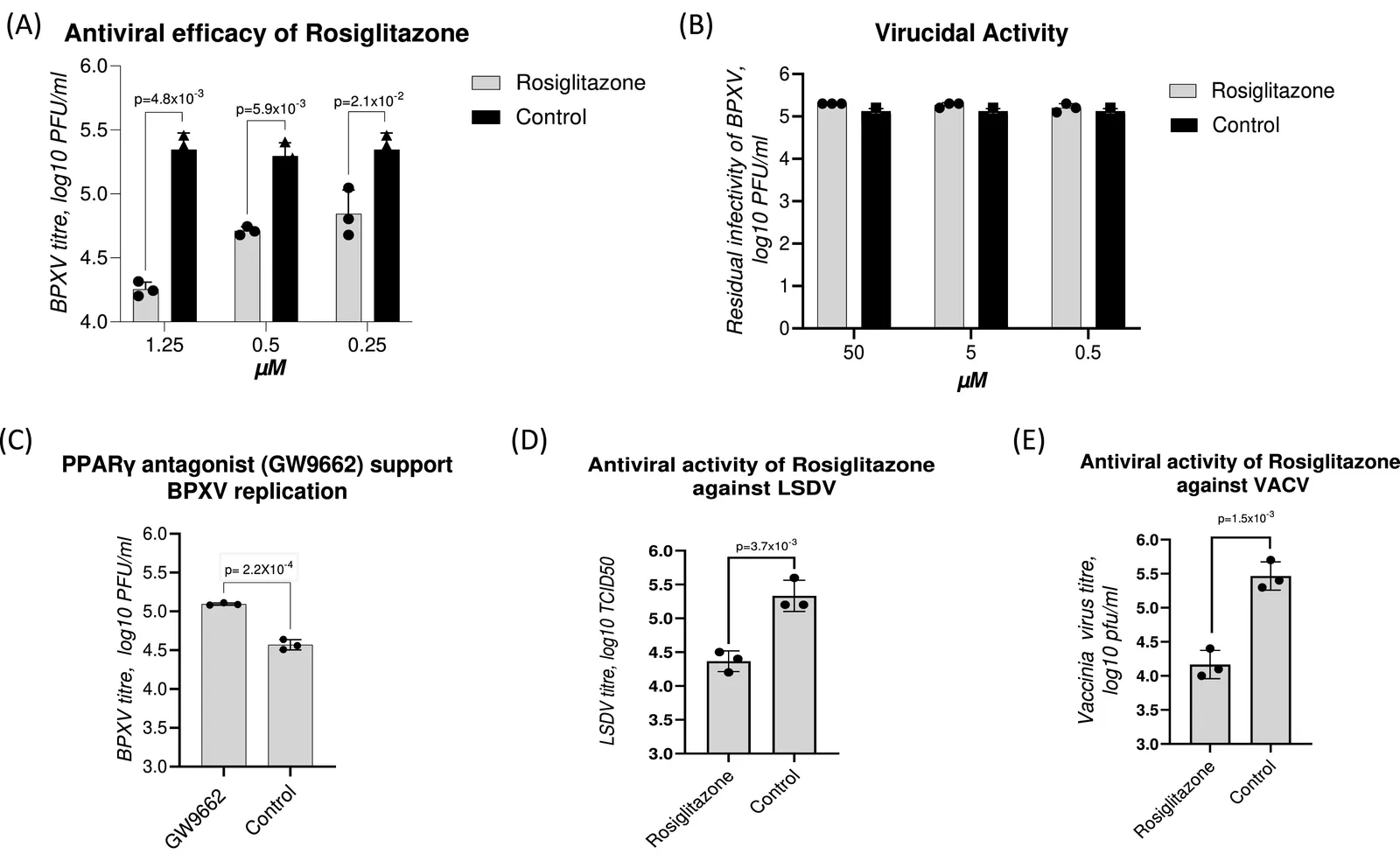
PPARγ antagonize BPXV replication. **(*A) Antiviral efficacy of Rosiglitazone.*** Vero cells, in triplicates, were infected with BPXV at MOI of 0.1 in the presence of indicated concentrations of Rosiglitazone or DMSO. The quantity of virus particles present in the cell culture supernatants at 48 hpi were determined using plaque assay. Results represent mean ± SD from three independent experiments. Pair-wise statistical comparisons were performed using Student’s t-test. ***(B) Virucidal activity.*** BPXV was incubated with indicated concentrations of Rosiglitazone or DMSO for 90 min at 37 °C. After that, the residual viral infectivity was determined by plaque assay. ***(C) PPARγ antagonist (GW9662) facilitate BPXV replication*.** Vero cells, in triplicates, were infected with BPXV at MOI of 0.1 in the presence of indicated concentrations of GW9662 or DMSO. The quantity of virus particles present in the cell culture supernatants at 48 hpi were determined by plaque assay. Results represent mean ± SD from three independent experiments. Pair-wise statistical comparisons were performed using Student’s t-test. **(*D-E) Effect of Rosiglitazone on LSDV and VACV replication.*** Vero cells, in triplicates, were infected with BPXV at MOI of 0.1 in the presence of indicated concentrations of Rosiglitazone or DMSO. The quantity of LSDV and Vaccinia virus particles present in the cell culture supernatants at 48 hpi were determined by plaque assay. Error bars indicate SD. n = 3 independent experiments. Pair-wise statistical comparisons were performed using the Student's *t*-test.

### 3.2 PPARγ signaling pathway interfere with BPXV protein synthesis

To examine which specific steps of the BPXV life cycle are affected by rosiglitazone, a time-of-addition assay was conducted. Rosiglitazone treatment resulted in comparable inhibition when applied pre-infection or at 1 hpi, 3 hpi, 6 hpi, 12 hpi, 18hpi and 24 hpi (Fig. 2**A**), indicating no impact on early viral processes such as attachment or entry. Notably, addition at 12 hpi resulted in only marginal impairment of BPXV replication, whereas addition after 18 hpi had no effect, suggesting that rosiglitazone potentially affect post-entry steps without affecting later stages such as virion assembly or release. The kinetic analysis of BPXV demonstrated that viral mRNA levels increased above the threshold by 3 h post-infection (hpi) and reached their peak at 18 hpi (**Fig. S2A**), while DNA synthesis started at 6 hpi and peaked at 24 hpi (**Fig. S2B**). The rosiglitazone treatment was shown to significantly reduce viral protein levels (Fig. 2**B-i** and **B-ii**) and marginally decreases viral DNA levels (Fig. 2**C**), without affecting, attachment (**Fig. S2D**), entry (**Fig. S2E**), viral mRNA synthesis (Fig. 2**D**) and release (**Fig. S2F**) in virus-infected cells. These results, together with our earlier findings, demonstrate that PPARγ inhibition specifically impairs BPXV protein synthesis and to a lesser extent viral DNA synthesis, while having minimal or no effect on other stages of the viral replication cycle.

**Fig. 2:**
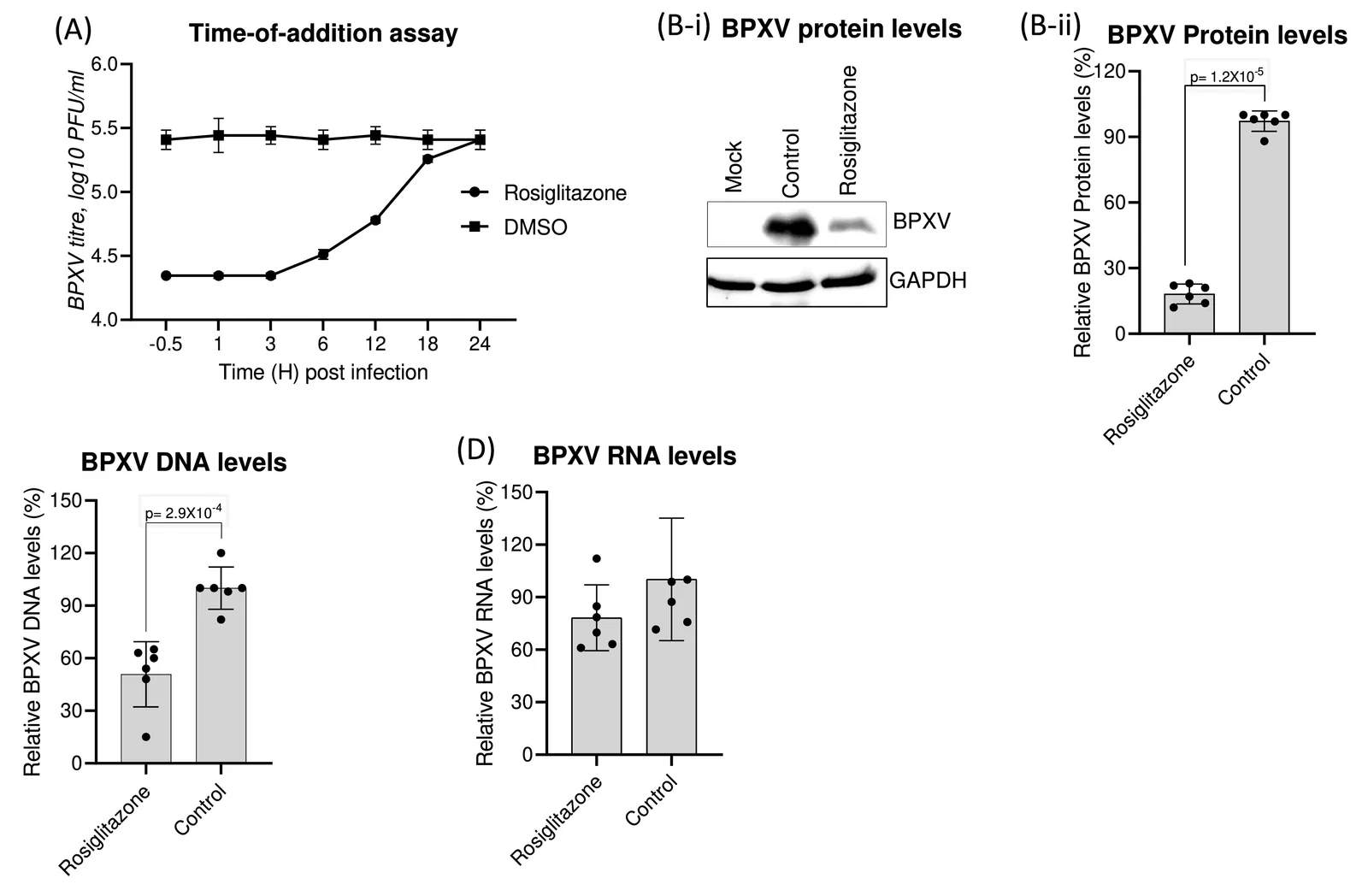
PPARγ agonist blocks BPXV protein and DNA synthesis. ***(A) Time-of-addition assay:*** Confluent monolayers of Vero cells, in triplicate, were infected with BPXV at MOI of 5 for 1 h, washed 5 times with PBS, and fresh medium with either rosiglitazone or DMSO was added at the indicated times. The supernatant was collected at 48 hpi and quantified by plaque assay. Error bars indicate SD. n = 3 independent experiments. Pair-wise statistical comparisons were performed using Student’s *t*-test. ***(B) BPXV protein levels*.** Vero cells, in triplicates, were infected with BPXV at 5 MOI, followed by washing with PBS and addition of fresh medium. Rosiglitazone or vehicle-controls were applied at 3 hpi. The cell lysates were prepared at 24 hpi. The levels of viral (upper panel) and house-keeping control protein (GAPDH) in lower panel were determined by Western blot analysis (**B-i**). The blots were quantified by densitometry (ImageJ) and the histogram shows the band intensity of the protein (**B-ii**). ***(C) BPXV DNA levels.*** Vero cells, in triplicates, were infected with BPXV (5 MOI), followed by washing with PBS and addition of fresh medium. Rosiglitazone or vehicle-controls were applied at 3 hpi. The cells were scraped at 24 hpi to isolate DNA. The BPXV M gene in both Rosiglitazone -treated and vehicle-control-treated Vero cells was quantified using qRT-PCR. Ct values were normalized with the housekeeping control gene (GAPDH), and relative fold change was calculated by the ∆∆ Ct method. n = 3 independent experiments. Error bars indicate SD. Pair-wise statistical comparisons were performed using Student’s *t*-test. ***(D) BPXV mRNA levels***. Vero cells, in triplicates, were infected with BPXV (5 MOI), followed by washing with PBS and addition of fresh medium. Rosiglitazone or vehicle-controls were applied at 4 hpi. Cells were scraped at 12 hpi to isolate RNA. cDNA was synthesized using oligo(dT) primers and quantified for BPXV M gene by qRT-PCR. Ct values were normalized with the GAPDH and relative fold change was calculated by the ∆∆ Ct method. n = 3 independent experiments. Error bars indicate SD. Pair-wise statistical comparisons were performed using Student’s *t*-test.

### 3.3 Dynamic interplay between miR-27a and PPARγ regulates BPXV mRNA translation

An upstream negative regulator of PPARγ, miR-27a (Chen et al., 2019b; Kim et al., 2010b), was also evaluated for its potential role in supporting viral replication. miR-27a is known to repress PPARγ by binding to the 3′ UTR of PPARγ mRNA (Fig. 3**A**). The expression kinetics of miR-27a and PPARγ mRNA revealed a negative correlation in the BPXV-infected cells. In the early phase (0 hpi to 6 hpi), miR-27a expression was on its lower level while PPARγ mRNA was on its peak (Fig. 3**B** and **C**). Notably, at 12 hpi, a time point corresponding to the peak of BPXV mRNA expression and translation, the miR-27a levels were at their highest levels while PPARγ mRNA levels were markedly reduced (Fig. 3**B** and **C**). Since Rosiglitazone treatment primarily impairs BPXV mRNA translation, which is presumed to occur between 6 and 12 hpi, this negative correlation between miR-27a and PPARγ during this critical window underscores a key regulatory axis controlling viral mRNA translation.

**Fig. 3:**
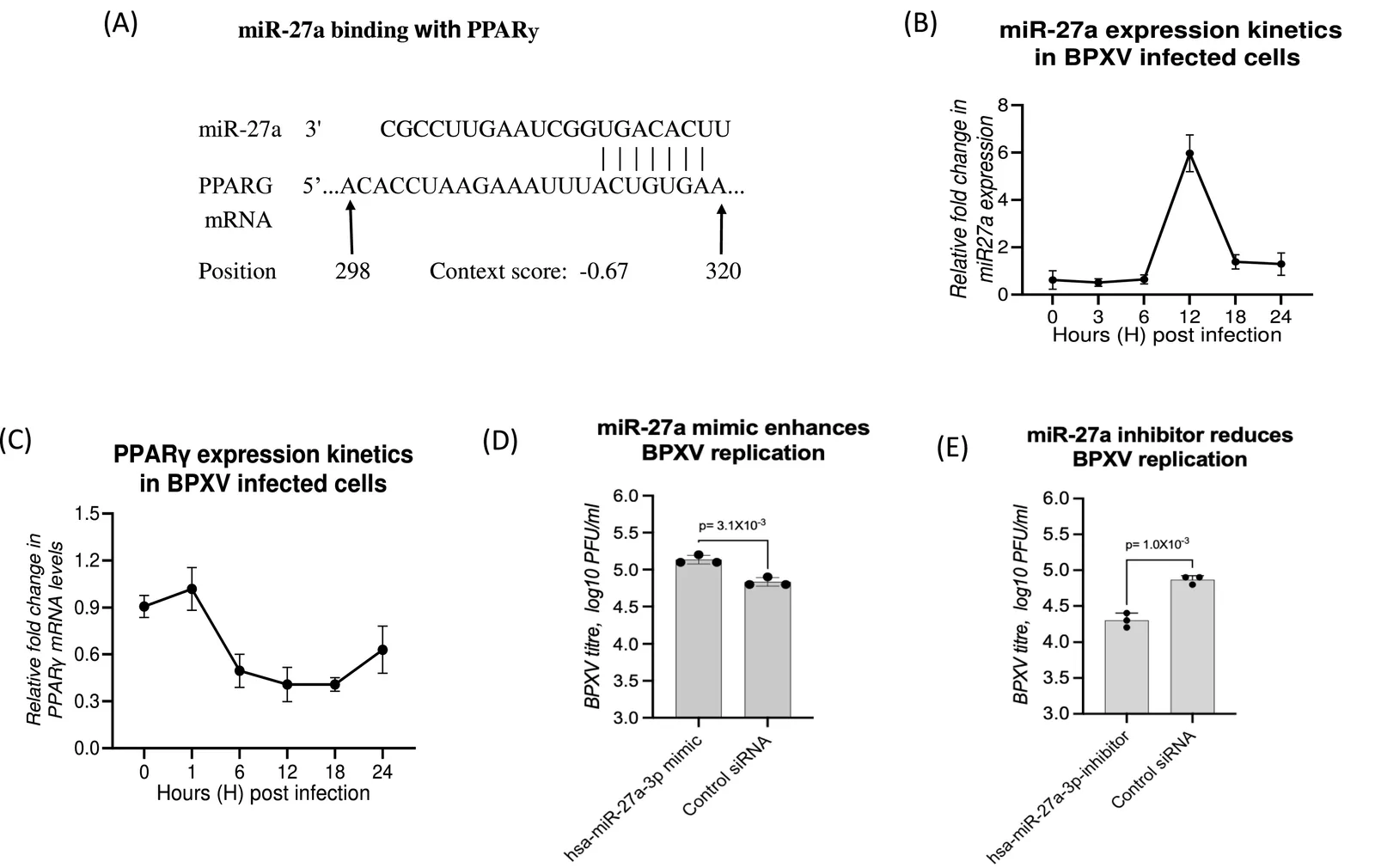
Dynamic interplay between miR-27a and PPARγregulates BPXV mRNA translation. ***(A). miR-27a binding with PPARγ:*** The miR-27a binding sites on 3′-UTR of PPARγ mRNA was identified by pairing the miRNA seed region and complementary sites within target mRNAs using TargetScan. The context score and position of miR-27a binding to 3′-UTR of PPARγ mRNA is shown. ***(B) miR-27a expression kinetics in BPXV infected cells:*** Vero cells, in triplicates, were infected with BPXV (5 MOI), followed by washing with PBS and addition of fresh medium. At the indicated time points, cells were harvested and total RNA was extracted. Following poly(A) tailing and cDNA synthesis, qRT-PCR was performed using a miR-27a-specific forward primer and a universal adaptor reverse primer. Expression levels were normalized by U6, housekeeping control microRNA and relative fold change was calculated by the ∆∆ Ct method. Results represent mean ± SD from three independent biological replicates. ***(C) PPARγ expression kinetics in BPXV infected cells:*** Vero cells, in triplicates, were infected with BPXV at 5 MOI, followed by washing with PBS and addition of fresh medium. Cells were scrapped at indicated time points and subjected for the quantification of PPARγ mRNA levels by qRT-PCR. Ct values were normalized with the GAPDH (housekeeping control gene) and relative fold change was calculated by the ∆∆ Ct method. Results represent mean ± SD from three independent experiments. Pair-wise statistical comparisons were performed using Student’s t-test. **(D) *miR-27a mimic enhances BPXV replication:*** Vero cells, in triplicates, were transfected with either hsa-miR-27a-3p mimic or control scrambled miRNA, followed by infection with BPXV at MOI of 0.1. At 48 hpi, culture supernatants were collected and viral titers were measured by plaque assay. Results represent mean ± SD from three independent experiments. Pair-wise statistical comparisons were performed using Student’s *t*-test. **(E) *miR-27a inhibitor reduces BPXV replication:*** Vero cells, in triplicates, were transfected with either hsa-miR-27a-3p inhibitor or control scrambled miRNA, followed by infection with BPXV at MOI of 0.1. At 48 hpi, culture supernatants were collected and viral titers were measured by plaque assay. Results represent mean ± SD from three independent experiments. Pair-wise statistical comparisons were performed using Student’s *t*-test.

Moreover, to establish a functional relationship between miR-27a and BPXV replication, we further investigated this relationship using miR-27a inhibitor. As shown in Fig. 3**D,** overexpression of miR-27a using a hsa-miR-27a-3p mimic significantly enhanced BPXV replication. Conversely, inhibition of endogenous miR-27a with hsa-miR-27a-3p inhibitor significantly reduced the viral titre (Fig. 3**E**). Taken together, these findings further demonstrate the virus-supportive role of miR-27a and support its involvement in promoting BPXV replication, potentially through the regulation of PPARγ.

### 3.4 PPARγ agonist interfere with BPXV replication via disrupting p38α MAPK–MNK1–eIF4E signaling pathway

Depending on the nature of the stimulus and the cellular context, p38 MAPK can function upstream to regulate PPARγ activity through phosphorylation-dependent mechanisms that influence its transcriptional activity, stability, or cofactor recruitment (Chen et al., 2019b; Schild et al., 2006). Conversely, in other settings, activated PPARγ can modulate p38 MAPK signaling by altering the expression of upstream kinases, phosphatases, or inflammatory mediators (Hua et al., 2022). This bidirectional cross-talk highlights a dynamic regulatory axis in which p38 MAPK and PPARγ fine-tune metabolic and inflammatory responses in a context-dependent manner (Hua et al., 2022). Since, BPXV mRNA translation occurs via a cap-dependent mechanism (Kumar et al., 2018a), in which the p38α MAPK–MNK1–eIF4E signaling pathway plays a vital role (Kumar et al., 2018b), we investigated whether there is an association between PPARγ and p38α or not. As shown in Fig. 4**A**, rosiglitazone treatment reduced the levels of phosphorylated p38α, MNK-1 and eIF4E compared to control-treated cells. Given that p38α-MNK1-eIF4E pathway is critical for viral mRNA translation within viral factories (Kumar et al., 2022), we next investigated whether the PPARγ antagonize localization of eIF4E in viral factories or not. As shown in Fig. 4**B**, rosiglitazone-treated cells exhibited a marked reduction in colocalization of eIF4E with viral factories, accompanied by decreased BPXV protein synthesis. Moreover, as shown in Fig. 5**A,** the antiviral effect of rosiglitazone was markedly attenuated in p38α-knockout cells, indicating that its antiviral activity is largely dependent on intact p38α signaling. In co-immunoprecipitation (Co-IP) assay, we do not observed any direct interaction between PPARγ and p38α. Since PPARγ does not directly interact with p38α MAPK, we further investigated the potential mechanism through which it regulates p38α signaling. As shown in Fig. 5B, PPARγ significantly upregulated the transcription of DUSP1, a MAPK phosphatase known to dephosphorylate and inactivate p38α MAPK (Chen et al., 2019a; Ferguson et al., 2016; Hammer et al., 2006b; Turpeinen et al., 2011). Moreover, treatment of DUSP1 inhibitor **(Fig S4)** alone did not exhibit any proviral effect (Fig. 5**C**). However, the DUSP1 inhibitor rescued BPXV replication in rosiglitazone treated cells (Fig. 5**C**). These findings suggest that PPARγ restricts BPXV replication by inducing DUSP1–mediated inhibition of p38α MAPK, thereby disrupting eIF4E-dependent viral mRNA translation at perinuclear replication sites.

**Fig. 4:**
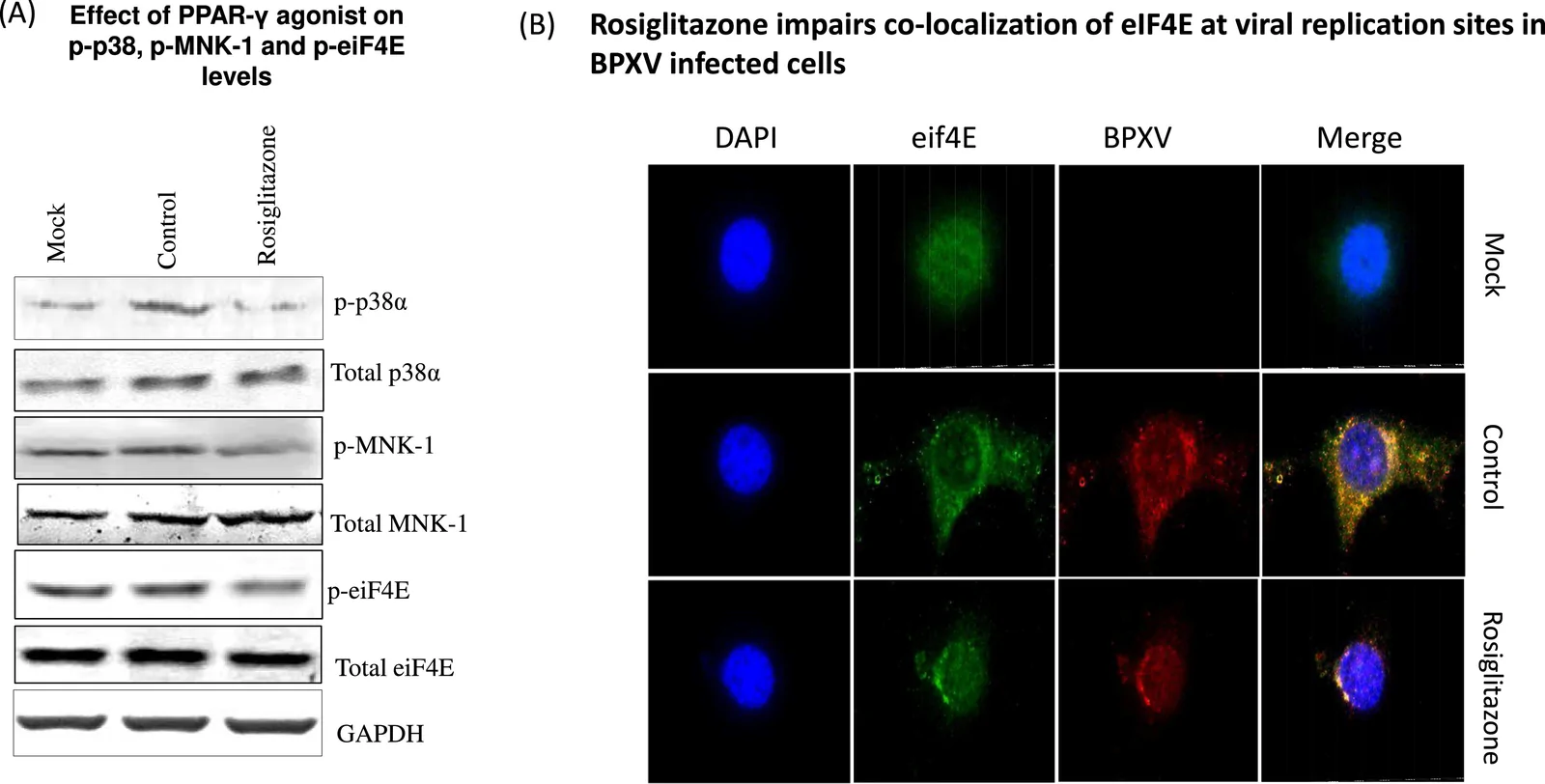
PPARγ regulates BPXV mRNA translation via modulating p38α MAPK–MNK1–eIF4E signaling pathway. ***(A) Effect of PPARγ agonist on p-p38α, p-MNK-1 and p-eIF4E levels.*** Vero cells, in triplicates, were infected with BPXV (5 MOI), followed by washing with PBS and addition of fresh medium. At 3 hpi, cells were treated with rosiglitazone or DMSO as a control. At 12 hpi, cells were collected and the levels of phosphorylated p38α, MNK1 and eIF4E were assessed by western blot analysis. ***(B) Rosiglitazone impairs co-localization of eIF4E at viral replication sites in BPXV infected cells.*** Vero cells were cultured in chamber slides and infected with BPXV at MOI of 5 for 1 h. Rosiglitazone was added at 4 hpi. At 36 hpi, BPXV proteins labeled with Rhodamine and host protein eIF4E labeled with FITC were detected by immunofluorescence.

**Fig. 5:**
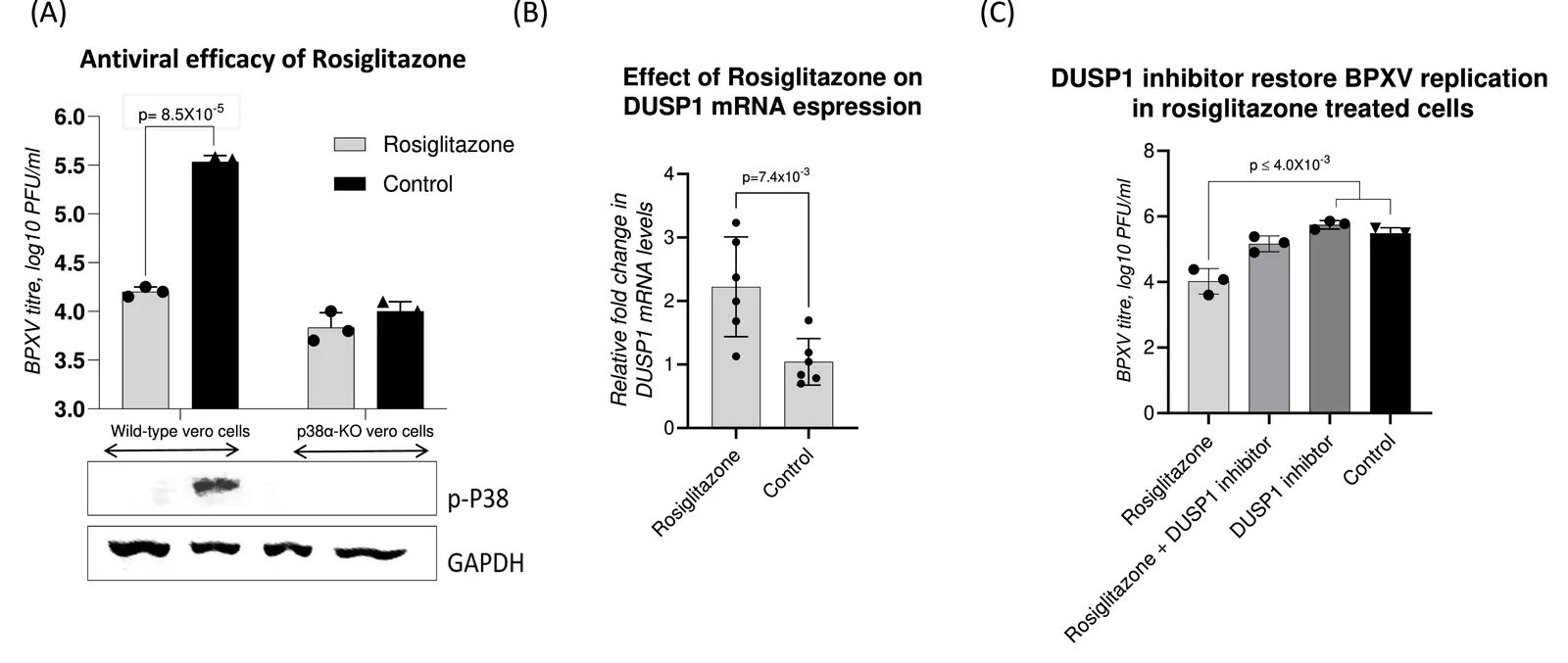
PPARγ promotes DUSP1 expression to negatively regulate p38α MAPK Signaling. ***(A) Antiviral efficacy of Rosiglitazone in p38α-KO Vero cells.*** p38α-KO Vero cells and wild-type Vero cells, in triplicates, were infected with BPXV (0.1 MOI), in presence of rosiglitazone or DMSO. The quantity of virus particles present in the p38α-KO Vero cells and wild-type vero cell supernatants at 48 hpi were determined by plaque assay. Results represent mean ± SD from three independent experiments. Pair-wise statistical comparisons were performed using Student’s t-test. ***(B) Effect of Rosiglitazone on DUSP1 mRNA expression:*** Vero cells, in triplicates, were infected with BPXV at 5 MOI, followed by washing with PBS and addition of fresh medium. Rosiglitazone was added at 4 hpi. Cells were scrapped at 18 hpi and subjected for the quantification of DUSP1 mRNA levels by qRT-PCR. Ct values were normalized with the GAPDH housekeeping control gene and relative fold change was calculated by the ∆∆ Ct method. n = 3 independent experiments. Error bars indicate SD. Pair-wise statistical comparisons were performed using Student’s *t*-test. ***(C) DUSP1 treatment rescue BPXV replication in Rosiglitazone treated cells.*** Vero cells, in triplicates, were infected with BPXV at MOI of 0.1 in the presence of Rosiglitazone, DUSP1 inhibitor, Rosiglitazone + DUSP1 inhibitor or DMSO. The quantity of virus particles present in the cell culture supernatants after 48 hpi were determined using a plaque assay. Error bars indicate SD. n = 3 independent experiment. Pair-wise statistical comparisons were performed using the Student's *t*-test.

### 3.5 In ovo antiviral efficacy of rosiglitazone against BPXV

Members of the family *Poxviridae* can infect chicken embryos and produce distinctly visible pock lesions on the CAM of the embryonated chicken eggs (Chander et al., 2022; Khandelwal et al., 2017; Kumar et al., 2024, 2018a). We utilized this model to evaluate the *in ovo* efficacy of rosiglitazone against BPXV. First, we determined the LD_50_ of Rosiglitazone: as shown in Fig. 6**A**, embryonic mortality was observed at concentrations ≥1.25 μg/egg, but not ≤0.31 μg/egg. The LD_50_ of rosiglitazone was found to be 1.64 μg/egg. Subsequently, embryonated eggs were infected with BPXV at 100 EID_50_, with or without rosiglitazone at the indicated concentrations (Fig. 6**B**). The rosiglitazone conferred protection against BPXV-associated mortality in a dose-dependent manner (Fig. 6**B**), with an EC_50_ of 0.26 μg/egg. Compared to the vehicle control, no obvious pock lesions were observed in eggs treated with rosiglitazone (protected groups) (Fig. 6**C**). Taken together, rosiglitazone treatment effectively prevents the development of BPXV-induced pock lesions on the CAM and the associated embryonic mortality, suggesting that activation of PPARγ signaling plays an antiviral role against BPXV.

**Fig. 6:**
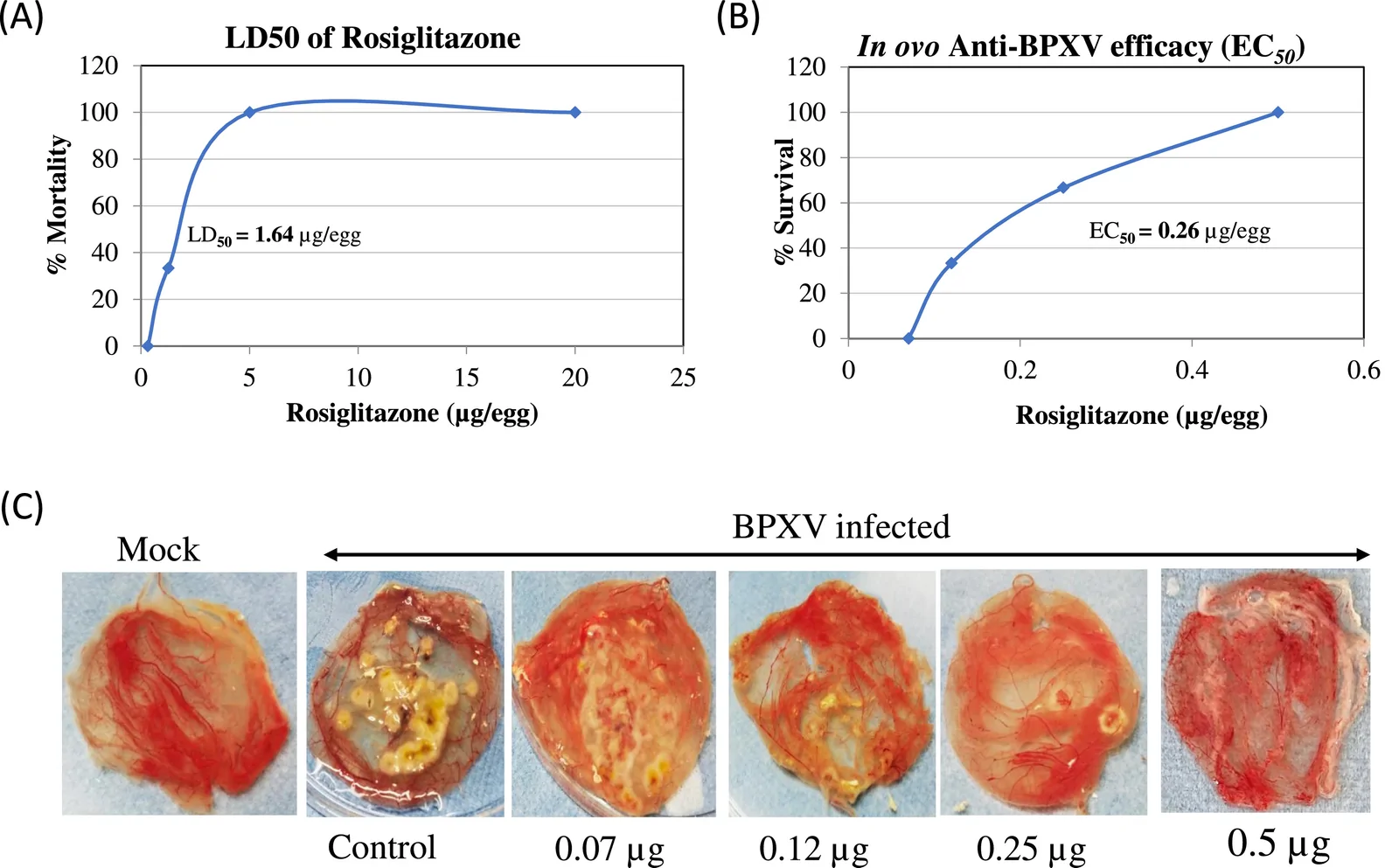
*In ovo* antiviral efficacy of Rosiglitazone against BPXV. ***(A)* LD_50_*determination:*** Varying concentrations of rosiglitazone were administered in triplicate into 10-day-old embryonated SPF chicken eggs, and embryo survival was monitored for five days post-inoculation. The LD_50_ was calculated to be 1.64 μg/egg using the Reed-Muench method. ***(B) In ovo Anti-BPXV efficacy* (EC_50_).** Five-fold serial dilutions of rosiglitazone or DMSO (control) were inoculated in triplicate via the CAM route into 10-day-old SPF eggs, followed by infection with 100 EID_50_ of BPXV. Embryo viability was assessed over six days, and the EC_50_ was determined to be 0.26 μg/egg using the Reed-Muench method. ***(C) CAM pock lesions:*** Pock lesions on CAM at various drug regimes.

### 3.6 PPARγ does not select drug-resistant BPXV variants

To evaluate the potential development of drug-resistant virus variants, BPXV was serially passaged 50 times in the presence of rosiglitazone or vehicle control (DMSO). At passage 50 (P50), both rosiglitazone-passaged and control-passaged viruses were assessed for their sensitivity to rosiglitazone. Similar to the virus at passage 0 (P0), both P50-rosiglitazone and P50-control viruses exhibited comparable susceptibility to rosiglitazone (Fig. 7**A**), showing approximately 7–8 fold reduction in viral titer upon treatment (Fig. 7**B**). These findings indicate that long-term exposure to rosiglitazone does not lead to the emergence of drug-resistant BPXV variants. However, in comparison to the virus at passage 0 (P0), both the P50-rosiglitazone and P50-control viruses showed increased replication (approximately 1 log higher in titer) (Fig. 7**A**) and generated larger plaques (Fig. 7**C**). This enhancement is likely due to an overall increase in viral fitness acquired through serial passaging.

**Fig. 7:**
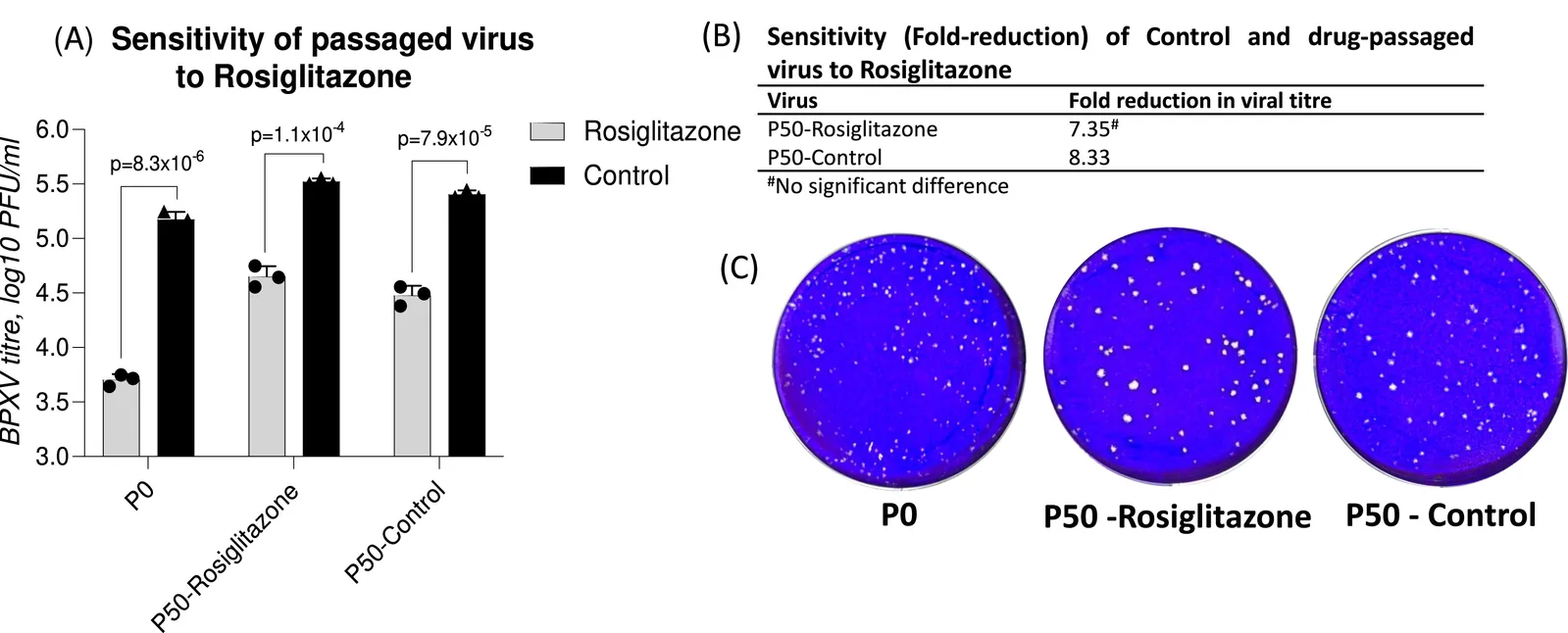
Evaluation of antiviral drug resistance against rosiglitazone. ***(A) Sensitivity of passaged virus to rosiglitazone.*** BPXV was sequentially passaged (P) 50-times in the medium containing 0.15 µM of rosiglitazone or equivalent volumes of DMSO. The original virus stock (P0), P_50_-rosiglitazone and P_50_-control viruses were used to infect Vero cells (n = 3) at an MOI of 0.1 and treated with either 0.45 µM of rosiglitazone or equivalent volumes of DMSO. The virus released in the supernatant at 48 hpi was quantified by plaque assay. Error bars indicate SD. Pair-wise statistical comparisons were performed using Student’s t-test. (B) Sensitivity (Fold-reduction) of control-and drug-passaged virus to rosiglitazone. (C) Plaque morphology of p50-control and drug-passaged virus.

## 4 Discussion

Recent studies have revealed that PPARγ can markedly influence the outcomes of viral infections (Layrolle et al., 2021). Depending on the specific virus, PPARγ has been reported to exert dual roles, wherein it can either promote or restrict viral infection (Layrolle et al., 2021; Potula et al., 2008; Wakui et al., 2010). However, the role of PPARγ in poxvirus infections, particularly in BPXV remains largely unexplored. A targeted screen using a kinase and phosphatase inhibitor library revealed that activation of PPARγ with the selective agonist rosiglitazone suppresses BPXV replication without exerting direct virucidal activity, whereas inhibition of PPARγ with GW9662 enhanced viral replication indicating that PPARγ functions as a host restriction factor for BPXV. Notably, rosiglitazone also inhibited LSDV and VACV replication, suggesting its broad-spectrum antiviral activity against multiple members of the *Poxviridae* family.

The time-of-addition and step-specific assays demonstrated that rosiglitazone treatment significantly impairs viral protein synthesis and marginally reduces viral DNA synthesis, while having minimal or no effect on other stages of the BPXV replication cycle, including viral attachment, entry, mRNA synthesis and release. To further investigate the role of PPAR-γ, we examined miR-27a, a well-characterized negative regulator of PPAR-γ expression (Li et al., 2019; Zhang et al., 2019). Consistent with previous studies, we observed an inverse relationship between miR-27a expression and PPAR-γ mRNA levels during BPXV infection (Bayati et al., 2022; Chen et al., 2019b). Since rosiglitazone treatment primarily impairs BPXV mRNA translation, which is presumed to occur between 6 and 12 hpi, this inverse correlation between miR-27a and PPARγ during this critical window underscores miR-27a–PPARγ as a key regulatory axis controlling viral mRNA translation. Complementary gain- and loss-of-function experiments using a hsa-miR-27a-3p mimic and hsa-miR-27a-3p inhibitor provided functional support for a proviral role of miR-27a, although the magnitude of the effect observed following miR-27a inhibition and/or overexpression was relatively modest. Previous studies have shown that miR-27a and PPARγ differentially regulate cytomegalovirus replication, with miR-27a acting as an antiviral factor during MCMV infection and PPARγ promoting HCMV replication (Buck et al., 2010; Rauwel et al., 2010). In contrast, our findings in BPXV infection demonstrate an opposite pattern, with miR-27a exhibiting a proviral role and PPARγ functioning as an antiviral factor, underscoring a virus-specific divergence in the miR-27a–PPARγ regulatory axis.

PPARγ can modulate inflammatory and antiviral signaling pathways through cross-talk with MAPK cascades, including the p38 MAPK pathway (Chen et al., 2019b; Glass and Saijo, 2010; Hua et al., 2022; Schild et al., 2006). In the present study, we observed that activation of PPARγ significantly suppressed BPXV mRNA translation and consequently reduced viral protein synthesis. Since poxviral mRNA translation predominantly occurs through a cap-dependent mechanism involving the p38α MAPK–MNK1–eIF4E signaling pathway (Chander et al., 2022), we investigated whether cross-talk exists between PPARγ signaling and the p38α MAPK–MNK1–eIF4E signaling pathway in BPXV-infected cells. Interestingly, rosiglitazone treatment markedly reduced the phosphorylation of p38α MAPK, MNK1, and eIF4E in BPXV-infected cells, eventually leading to a substantial reduction in viral protein synthesis. Moreover, immunofluorescence analysis revealed that rosiglitazone treatment reduced the accumulation of eIF4E within poxvirus replication factories, which are sites associated with viral mRNA translation (Kumar et al., 2022a; Walsh, 2010). These findings suggest that PPARγ activation interferes with the p38α MAPK–MNK1–eIF4E signaling pathway and thereby contributes to the suppression of BPXV protein synthesis.

Furthermore, rosiglitazone significantly decreased BPXV titers in wild-type cells but failed to inhibit viral replication in p38α-knockout cells. Although p38α deficiency itself impaired BPXV replication, the loss of the additional antiviral effect of rosiglitazone treatment in p38α-knockout cells supports the conclusion that p38α MAPK signaling is required, at least in part, for the antiviral activity mediated by PPARγ. Importantly, the persistence of residual BPXV replication following p38α depletion may reflect the contribution of other cellular factors or compensatory signaling pathways that can partially sustain viral replication in the absence of p38α (Chander et al., 2022). Previous studies have suggested that other p38 isoforms, particularly p38γ, as well as alternative MAPK pathways such as ERK, may contribute to poxvirus replication (Chander et al., 2022; Kumar et al., 2020). Thus, these pathways could potentially contribute to the residual replication observed in p38α-knockout cells. However, p38γ and ERK signaling were not directly investigated in the present study, and their contribution to residual BPXV replication therefore remains a plausible but unconfirmed possibility.

These observations also emphasize that inhibition or genetic disruption of a single proviral factor is not necessarily expected to completely abolish viral replication (Cisneros et al., 2022). BPXV replication is a complex multistep process that depends on multiple host proteins and signaling pathways (Kumar et al., 2018a). Consequently, disruption of p38α may impair an important component of the viral translational machinery while allowing other cellular pathways to sustain residual viral replication (Chander et al., 2022). Further studies are required to comprehensively characterize the contribution of other p38 isoforms, ERK, and additional host signaling pathways to the residual BPXV replication observed in p38α-knockout cells.

PPARγ is a nuclear receptor that may modulate p38α MAPK signaling through either direct or indirect mechanisms (Brunmeir and Xu, 2018; Wang et al., 2015). however, the mechanism by which PPARγ activation influences p38α MAPK signaling in BPXV-infected cells remains unclear. Our immunoprecipitation assay did not reveal any direct interaction between PPARγ and p38α MAPK, suggesting that PPARγ regulates this signaling pathway through an indirect mechanism. We therefore explored the possibility that this regulation is mediated through upstream regulatory factors of p38α MAPK (Hammer et al., 2006a; Mo et al., 2018). One plausible mediator of this regulation is dual-specificity phosphatase-1 (DUSP1/MKP-1), a well-known negative regulator of MAPK signaling (Chen et al., 2019a; Ferguson et al., 2016; Hammer et al., 2006b; Turpeinen et al., 2011). Consistent with previous studies, we observed that PPARγ activation resulted in transcriptional upregulation of DUSP1 expression. Moreover, inhibition of DUSP1 restored BPXV replication in rosiglitazone-treated cells, providing functional support for the involvement of DUSP1 in the PPARγ-mediated antiviral response. Together, these findings support a model in which PPARγ activation promotes DUSP1-mediated attenuation of p38α MAPK signaling, thereby reducing downstream MNK1–eIF4E activity and impairing viral protein synthesis.

Collectively, the relationship between miR-27a and the PPARγ–DUSP1 pathway may therefore represent an additional layer of host regulation during BPXV infection. Increased miR-27a expression during the period of active viral translation may contribute to reduced PPARγ expression, thereby favoring signaling conditions that support p38α-dependent viral protein synthesis. Conversely, activation of PPARγ by rosiglitazone enhance DUSP1-mediated suppression of p38α signaling and consequently restrict BPXV protein synthesis.

Although our findings support a model in which rosiglitazone inhibits BPXV mRNA translation through the PPAR-γ–DUSP-1–p38α–MNK1–eIF4E signaling axis (Fig. 8), we cannot exclude the possibility that PPARγ-mediated metabolic reprogramming contributes to the observed antiviral phenotype (Montaigne et al., 2021; Pei et al., 2023; Rosen and Spiegelman, 2001; Varga et al., 2011; Zandi et al., 2025). In addition, the potential involvement of other PPAR isoforms, miR-27a targets, p38 isoforms, or alternative MAPK pathways remains to be determined. Future studies using selective genetic or pharmacological approaches will be necessary to define the relative contributions of these pathways to BPXV replication.

**Fig. 8:**
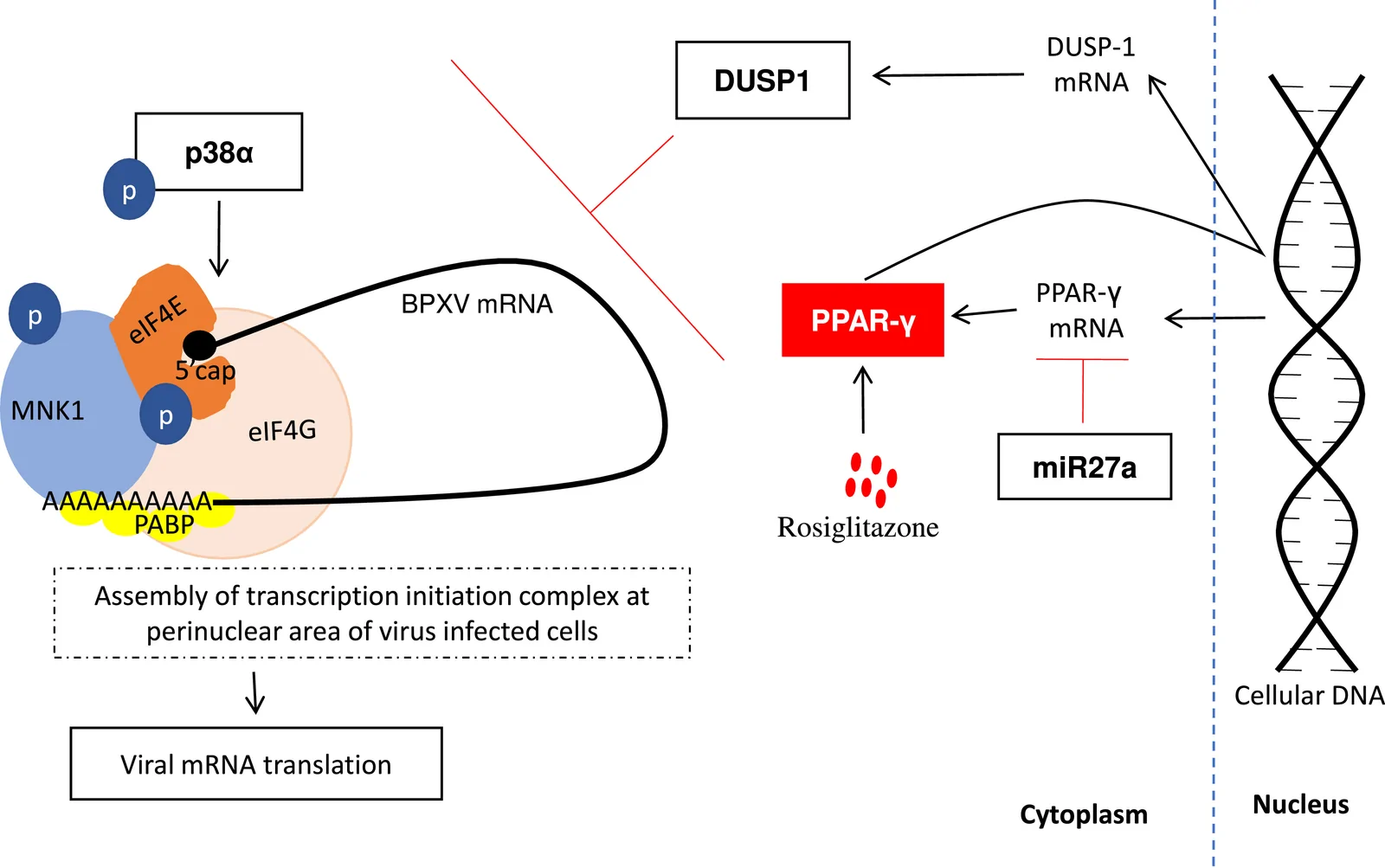
Interplay between miR27a and PPARγ - DUSP1 regulates BPXV mRNA translation via modulating p38α MAPK–MNK1–eIF4E signaling axis. The PPARγ agonist, rosiglitazone activates PPARγ signalling and interfere with BPXV mRNA translation via enhancing mRNA expression of DUSP1 that disrupts p38α MAPK–MNK1–eIF4E signaling axis. A microRNA, miR27a serve as proviral factor which binds with 3′-UTR of PPARG mRNA facilitates downregulation of PPARγ and subsequently facilitates viral mRNA translation or replication.

We also demonstrated that rosiglitazone conferred dose-dependent protection against BPXV-induced mortality and pock lesion formation in the embryonated chicken egg *in ovo* model, further confirming that activated PPARγ exerts a potent antiviral effect against BPXV in vivo system as well. Viruses rapidly develop drug-resistant variants; therefore, developing effective antiviral therapeutics is a major challenge (Khandelwal et al., 2017; Kumar et al., 2024). While direct-acting antiviral agents are well known to rapidly induce drug-resistant phenotypes, host-directed antiviral agents are less prone to promote the emergence of drug resistance (Kumar et al., 2023). Our findings demonstrate that long-term exposure of BPXV to rosiglitazone does not lead to the development of drug-resistant variants. After 50 serial passages in the presence of rosiglitazone or vehicle control, both virus populations remained equally sensitive to rosiglitazone, showing a consistent ∼10-fold reduction in viral titers upon treatment. However, both passaged viruses exhibited increased replication capacity (approximately 1 log higher viral titers) and formed larger plaques compared to the original (P0) virus, which may reflect enhanced overall viral fitness independently of rosiglitazone treatment, without compromising the antiviral efficacy of the drug (Kumar et al., 2022a). These findings suggest that the increased fitness acquired during serial passage did not result in an apparent loss of susceptibility to rosiglitazone, consistent with the proposed host-directed nature of its antiviral activity.

In conclusion, we demonstrate for the first time that the dynamic interplay between miR-27a and PPARγ-DUSP1 during BPXV replication regulates viral mRNA translation via the p38α MAPK–MNK1–eIF4E cellular signaling pathway (Fig. 8). This mechanism underscores a promising host-directed antiviral strategy targeting cellular signaling pathways essential for viral protein synthesis and offers new avenues for therapeutic intervention against poxviruses.

## Declaration of generative AI and AI-assisted technologies in the writing process

During the preparation of this work the author(s) used ChatGPT in order to improve English language only. After using this tool/service, the author(s) reviewed and edited the content as needed and take(s) full responsibility for the content of the publication.

## Funding

This work was supported by the Indian Council of Agricultural Research, New Delhi (grant number IXX14586 to N-Ku and NASF/ABA-8027/2020-21 to N-Ku).

## CRediT authorship contribution statement

**Garvit Kumar:** Writing – original draft, Validation, Methodology, Data curation. **Assim Verma:** Visualization, Methodology. **Shweta Dhanda:** Methodology. **Himanshu Kamboj:** Methodology. **Riyesh Thachamvally:** Resources, Methodology. **Shoor Vir Singh:** Supervision. **Ram Kumar:** Visualization, Methodology. **Naveen Kumar:** Writing – review & editing, Supervision, Funding acquisition, Formal analysis, Conceptualization.

## Declaration of competing interest

All authors have read the journal’s policy on disclosure of potential conflicts of interest and declared no conflict of interest.

## Data Availability

Data will be made available on request.
